# Patterns of Skin Disease among Prisoners in Central Prison in Nepal: A Descriptive Cross-sectional Study

**DOI:** 10.31729/jnma.8596

**Published:** 2024-05-31

**Authors:** Bikrant Dhakal, Paras Modi Pangeni, Prashanna Man Maharjan, Shrija Rijal, Subash Wagle, Neeraj Thapa, Bhagat Lal Shrestha

**Affiliations:** 1 Central Jail Hospital, Baghdurbar, Kathmandu, Nepal; 2 Central Department of Public Health, Institute of Medicine, Maharajgunj, Nepal; 3 Nepal Medical College, Jorpati, Nepal

**Keywords:** *dermatophytosis*, *fungi*, *skin disease*

## Abstract

**Introduction:**

Skin diseases pose a significant health challenge globally, especially within prison settings where overcrowding and inadequate sanitation create a breeding ground for infections. Prisons, as part of society, exist in a dynamic equilibrium, serving as potential sources of infections that can spread beyond their confines. Despite facing similar challenges, there is a dearth of research focusing on skin diseases among inmates in Nepal. This study aims to assess patterns of skin diseases among prisoners.

**Methods:**

This was a descriptive cross-sectional study at the central prison, Kathmandu from December 2023 to March 2024. All patients with skin disease visiting the consultant Dermatologist and giving consent were included in this study. Ethical approval was obtained from the Nepal Health Research Council. Data were entered in Microsoft Excel 2010 and analysis was done by the Statistical Package for the Social Sciences software. Data were presented in the form of frequency and percentage.

**Results:**

A total of 253 patients visited the consultant Dermatologist for skin conditions. Out of which, eczema was 67 (26.50%), fungal infections 57 (22.50%), and bacterial infections 51 (20.10%). The mean age of the respondents was 34.21±12 years.

**Conclusions:**

The fungal and bacterial infections were common among the prisoners.

## INTRODUCTION

Skin diseases are widespread in prisons due to overcrowding and poor sanitation. Prison inmates are among the vulnerable groups.^1,2^ In Nepal, like many other countries, prisons are often overcrowded and lack adequate healthcare facilities, making prisoners particularly vulnerable to various health conditions, including skin diseases.^3^ Despite the increasing vulnerability, the health issues of prisoners are understudied.^4^ Skin diseases can significantly affect a person's quality of life and more certainly overcrowding, hygiene limitations, and close contact in prisons elevate the risk of skin infections.^5^ Some of the common skin diseases that are prevalent among prison inmates are scabies, non-specific dermatitis, pediculosis, psoriasis, alopecia, acne, mycosis, eczema, skin infections, skin infestations, urticaria, hyperpigmentation, etc.^6,7^ In Nepal, very little work has been done on the study area that describes patterns of skin diseases among prison inmates. Therefore, this study aims to determine the pattern of skin diseases among prison inmates.

## METHODS

A descriptive cross-sectional study was conducted in a central jail, in Kathmandu, Nepal. All patients visiting consultant Dermatologist for skin conditions between December 2023 to March 2024 were included in the study. The patient over the age of 18 years were included in the study and those who had dermatological diseases diagnosed before coming to jail were excluded from the study. The ethical approval was obtained from the Ethical Review Board of the Nepal Health Research Council (Ref no 803). Written consent was taken from those willing to participate. The study possesses minimal risks during data collection due to the use of routine history taking and laboratory examinations of the skin. Prisoners were informed about the study through an information sheet (Nepali language) explaining the procedures: cutaneous examination, medical history, and a potential second visit for treatment.

The consultant dermatologist then conducted medical history interviews and cutaneous examinations at the jail hospital. Examinations included clinical descriptions of lesions, palpation, dermatoscopy, and KOH tests as needed. Data was recorded in dedicated skin examination forms. Prisoners requiring further consultation or treatment were invited for a voluntary second visit, where appropriate interventions and medication prescriptions were provided free of charge within the Central Jail Hospital. Data were entered in Microsoft Excel 2010 and analysis was done by the Statistical Package for the Social Sciences software. Data were presented in the form of frequency and percentage.

## RESULTS

Out of 253 participants, 127 (50.20)% of the population was under 30 years of age. The proportion of male respondents was 180 (71.10%) (Table 1).

**Table 1:** Socio-demographic details (n=253).

Characteristic	Category	n (%)
Age category	<30 Years	127 (50.20)
	>30 Years	126 (49.80)
Sex	Male	180 (71.10)
	Female	73 (28.90)
Address	Terai	84 (33.20)
	Hill	129 (50.98)
	Mountain	26 (10.27)
	Foreign	14 (5.53)
Occupation	Farmer	24 (9.48)
	Business	67 (26.48)
	Private job	115 (45.45)
	Government job	10 (3.95)
	Labour	2 (0.79)
	Others	35 (13.83)
Marital status	Unmarried	82 (32.41)
	Married	168 (66.40)
	Divorced	3 (1.18)
Education	Illiterate	18 (7.11)
	Primary level	70 (27.66)
	Secondary level	88 (34.78)
	High school	34 (13.43)
	Bachelors & above	43 (16.99)
Comorbidity status	No	165 (65.21)
	Yes	88 (34.79)
	Diabetes mellitus	23 (26.13)
	Hypertension	32 (36.36)
	Others	33 (37.50)
Smoking status	Non-smoker	117 (46.24)
	Smoker	136 (53.76)
Alcohol use	No	172 (67.98)
	Yes	81 (32.01)

Eczema accounted for 67 (26.48%)of cases, fungal infections 104 (41.10%) and bacterial infections 51 (20.15%). In regards to fungal infection, 47 (45.19%) cases of tinea was found. whereas dermatitis was responsible for 34 (13.43%) of cases (Table 2).

**Table 2:** Pattern of Skin Diseases (n=253).

Skin disease nature	n (%)
Eczema	67 (26.48)
Dermatitis	34 (13.43)
Lichen Simplex Chronicus	20 (7.90)
Fungal	104 (41.10)
Pityriasis	8 (3.16)
Bacterial	51 (20.15)
Acne	36 (14.22)
Autoimmune	21 (8.30)
Lipoma	6 (2.37)
Psoriasis	5 (1.97)
Alopecia	5 (1.97)
Viral	19 (7.50)
A Utricaria	13 (5.13)
Parasitic	17 (6.71)
Pigmentation	10 (3.95)
Melasma	7 (2.76)
Others	56 (22.13)

## DISCUSSION

Our study identified a higher proportion of fungal infections (41.10%) followed by eczematous diseases (26.48%). The pattern of skin diseases was similar when compared to studies conducted in Nepal, India, Pakistan, Taiwan, and Nigeria.^2,3,8,10,11^ Prison inmates may suffer from higher rates of eczema and fungal skin infections due to factors such as overcrowding, poor hygiene conditions, limited access to medical care, and increased stress levels.^3,4,8^ The sleeping area in the cells was far less than that of the dormitories, resulting in increased skin contact and more infections. According to data, the prison is more than twice as full as it can accommodate, meaning that 2750 inmates are housed in a space meant for 1250 inmates.^12^ The prevalence of infectious dermatoses is an index of socioeconomic development. Therefore, the highest burdens of transmissible dermatoses occur in resource-poor settings in developing countries. Information on the prevalence of skin infections among Nepali prison inmates was limited.

The predominant clinical manifestation was identified as eczema 67 (26.48%), tinea 47 (18.57%) followed by acne, dermatitis, scabies, pityriasis, etc. The health problems faced by prisoners aren't confined to prison alone; they also extend to society, as over 95% of inmates will reintegrate into the community eventually.^13^ Prisons and jails are constantly in flux with people entering and leaving, highlighting the importance of providing proper medical and social support for inmates.^14^ Taking care of inmates by ensuring access to medication, reducing overcrowding, and maintaining hygiene can lead to a decrease in skin disease rates inside prison.

Improving personal hygiene and living conditions can reduce the prevalence of infections. Crowded environments and sharing personal items are key factors in spreading infections and infestations. Basic healthcare services in prison should match the standards available in the community, and prison health professionals should receive adequate training to address skin conditions effectively. This study was carried out for a short duration therefore this study does not cover the disease that varies with climate which is the limitation of this study.

## CONCLUSIONS

The common skin conditions observed in prisoners were dermatitis, tinea, and acne. Most of the participants were male.
